# A Margin-of-Exposure Approach to Assessment of Noncancer Risks of Dioxins Based on Human Exposure and Response Data

**DOI:** 10.1289/ehp.11514

**Published:** 2008-06-16

**Authors:** Lesa L. Aylward, Julie E. Goodman, Gail Charnley, Lorenz R. Rhomberg

**Affiliations:** 1 Summit Toxicology, LLP, Falls Church, Virginia, USA; 2 Gradient Corporation, Cambridge, Massachusetts, USA; 3 HealthRisk Strategies, Washington, DC, USA

**Keywords:** benchmark dose, dioxin, enzyme induction, risk assessment, thyroid, tissue concentrations

## Abstract

**Background:**

Risk assessment of human environmental exposure to polychlorinated dibenzo-*p*-dioxins and dibenzofurans (PCDD/PCDFs) and other dioxin-like compounds is complicated by several factors, including limitations in measuring intakes because of the low concentrations of these compounds in foods and the environment and interspecies differences in pharmacokinetics and responses.

**Objectives:**

We examined the feasibility of relying directly on human studies of exposure and potential responses to PCDD/PCDFs and related compounds in terms of measured lipid-adjusted concentrations to assess margin of exposure (MOE) in a quantitative, benchmark dose (BMD)–based framework using representative exposure and selected response data sets.

**Methods:**

We characterize estimated central tendency and upper-bound general U.S. population lipid-adjusted concentrations of PCDD/PCDFs from the 1970s and early 2000s based on available data sets. Estimates of benchmark concentrations for three example responses of interest (induction of cytochrome P4501A2 activity, dental anomalies, and neonatal thyroid hormone alterations) were derived based on selected human studies.

**Results:**

The exposure data sets indicate that current serum lipid concentrations in young adults are approximately 6- to 7-fold lower than 1970s-era concentrations. Estimated MOEs for each end point based on current serum lipid concentrations range from < 10 for neonatal thyroid hormone concentrations to > 100 for dental anomalies—approximately 6-fold greater than would have existed during the 1970s.

**Conclusions:**

Human studies of dioxin exposure and outcomes can be used in a BMD framework for quantitative assessments of MOE. Incomplete exposure characterization can complicate the use of such studies in a BMD framework.

Exposure to dioxins and related compounds has been declining in the United States for over two decades in response to regulatory and other actions taken to reduce their generation and emissions into the environment (reviewed by Hays and Aylward 2003). Estimates of quantifiable emissions of polychlorinated dibenzo-*p*-dioxins and polychlorinated dibenzofurans (PCDD/PCDFs) and of dioxin-like polychlorinated biphenyls (PCBs) declined by a factor of 10 between 1987 and 2000, with the greatest reduction achieved through the control of municipal waste incineration [U.S. Environmental Protection Agency (EPA) 2006]. Overall emissions declines have been paralleled by severalfold reductions in estimates of dietary intake (Figure 1) and serum lipid–adjusted concentrations in the general population (reviewed by Hays and Aylward 2003; Lorber 2002).

The concomitant reduction in human health risks that have accompanied the declines in PCDD/PCDF/PCB exposure can be characterized by comparing past and present margins of exposure (MOEs) for different effects. MOEs are determined by dividing a point of departure (POD), derived from dose–response data, by relevant human exposure data. As exposure declines, MOEs become larger. MOEs do not reflect judgments about safety and are not themselves an indication of the likelihood of a risk. Interpreting MOEs in the context of risk assessment and risk management takes into account such factors as the slope of the dose–response relationship in the observable range, type of effect, mode of action, nature and extent of associated uncertainties, and human variation in susceptibility to the response of concern (Risk Commission 1997). In its recent draft risk assessment of dioxins, the U.S. EPA (2003) relied on an MOE approach to evaluate potential risks from PCDD/PCDFs based principally on animal data. The U.S. EPA has so far chosen not to establish a reference dose or other exposure limit for PCDD/PCDFs, and no acceptable daily intake has been established by the U.S. Food and Drug Administration.

Meanwhile, the U.K. Food Standards Agency (UKFSA), the Joint Food and Agriculture Organization/World Health Organization (FAO/WHO) Expert Committee on Food Additives (JECFA), and the European Commission’s Scientific Committee on Food (ECSCF) have established tolerable daily, weekly, or monthly intakes for combined exposures to PCDD/PCDFs based on the most sensitive end point observed in laboratory animals: developmental reproductive effects in male rats exposed prenatally to 2,3,7,8-tetrachlorodibenzo-*p*-dioxin (TCDD) [ECSCF 2001; JECFA 2001; United Kingdom Committee on Toxicology (UKCOT) 2001]. These efforts derived from earlier efforts by the WHO (1998), which pioneered the use of body burden for risk assessment of dioxins. Although health protective, those limits are derived from an animal model with questionable relevance to human health both qualitatively and quantitatively (Charnley and Kimbrough 2006; Connor and Aylward 2006). The U.S. EPA’s most recent draft risk assessment of PCDD/PCDFs relies primarily on laboratory animal data to calculate a series of MOEs for different effects (U.S. EPA 2003), raising important interspecies dose metric and toxicity extrapolation issues.

In the recent evaluations, both the ECSCF (2001) and JECFA (2001) noted that human data sets were insufficient for quantitative risk assessment. However, since these evaluations, a substantial body of literature examining human responses to dioxins using measurements of serum lipid concentration as the marker of exposure has developed. In this study we explored the use of human data sets on exposure and noncancer end points in the context of an MOE approach to risk assessment for PCDD/PCDFs and toxic equivalent (TEQ)–contributing PCB compounds. Exposure and response data in this effort are based on the most commonly used metric in such studies: serum lipid concentration of TCDD TEQs as estimated using the WHO toxic equivalency factors (TEFs) (van den Berg et al. 1998, 2006). Reliance on circulating serum lipid concentrations avoids issues associated with estimating body burden (estimates of body burden are highly influenced by assumptions regarding body fat content and degree of liver sequestration); provides an exposure metric that can be assessed directly (rather than calculated using assumptions); and is of high biological relevance to a variety of potential target tissue responses.

Exposure data are derived from several sources: analysis of adipose tissue samples collected from young adults of service age during the Vietnam era (including Vietnam veterans) during the 1970s (Kang et al. 1990); analysis of blood samples collected from a representative sample of the U.S. population from the National Health and Nutrition Evaluation Survey (NHANES) conducted in 2001–2002 [National Center for Health Statistics (NCHS) 2008]; and analysis of blood samples collected from residents of Michigan studied as part of an exposure evaluation conducted by the University of Michigan in 2005 [University of Michigan Dioxin Exposure Study (UMDES) 2008]. Exposures are compared among age groups and among birth cohorts. Using lipid-adjusted total dioxin serum TEQ values as the dose metric allowed us to compare internal doses based entirely on human data.

Example data sets on noncancer end points were chosen to demonstrate various approaches to using epidemiologic data in a quantitative risk assessment and on the basis of biological plausibility and interest in the data sets. End points include induction of the cytochrome P450 1A2 (CYP1A2) enzyme among highly exposed adults who accidentally ate contaminated rice oil in Taiwan (the Yucheng cohort) (Lambert et al. 2006); the occurrence of developmental dental defects among children highly exposed to TCDD as a result of the 1976 reactor vessel explosion at a chemical plant in Seveso, Italy (Alaluusua et al. 2004); and changes in thyroid hormone measures in infants exposed to background levels of PCDDs and PCBs in the Netherlands in the late 1980s and early 1990s (Koopman-Esseboom et al. 1994). In each case we defined a POD using an appropriate benchmark dose (BMD) approach (U.S. EPA 2000), expressing dose as serum lipid TEQ. We estimated current MOEs for those effects (the margins between the BMDs and measures of serum lipid TEQ in the general U.S. population) and discuss the change in MOE compared to 1970s exposures.

The objective of this study was to *a*) provide an assessment of the degree of change in lipid-adjusted TEQ concentrations over the past three decades, and *b*) demonstrate the use of example human data sets in an MOE framework for assessing noncancer risks of dioxins and related compounds. A comprehensive assessment of the weight of the evidence for the selected end points and within the full body of available human data was not conducted and was outside the scope of this effort. However, implementation of this MOE approach in a comprehensive risk assessment for dioxins would include a weight-of-evidence assessment as a critical component.

## Methods

### Exposure characterization

For this analysis, we evaluated three data sets to estimate serum lipid concentrations of PCDDs and PCDFs. The U.S. EPA conducted the National Human Adipose Tissue Survey (NHATS) from 1970 to 1987 to monitor chemicals in adipose tissue in a statistically representative sample of U.S. residents. Seventeen PCDD/PCDFs were measured in a subset of NHATS tissue sampled between 1971 and 1982 from 36 Vietnam veterans, 79 non-Vietnam veterans, and 80 civilian men who were born between 1936 and 1954 and were between 20 and 45 years of age at the time of sampling (this subset was not necessarily statistically representative) (Kang et al. 1990). Because no differences in any congener concentrations were found among these groups, we used the entire study group of 195 in the present to characterize adipose tissue lipid-adjusted concentrations of PCDD and PCDF compounds during the 1970s. No PCB compounds were measured in that study. We assumed that the lipid-adjusted adipose tissue concentrations measured in this study reflect the lipid-adjusted concentrations in serum in these individuals (Patterson et al. 1988).

To determine how congener levels have changed over time, we compared the results from the 1971–1982 samples analyzed by Kang et al. (1990) with recent data from two data sets. The first data set comprises measurements from serum collected for NHANES 2001–2002 (NCHS 2008) and analyzed for 17 PCDD/PCDFs from a subsample of participants. That study used a complex, multistage, probability sampling design to select participants representative of the civilian, noninstitutionalized U.S. population. Using analytical guidelines for these data provided by the National Center for Health Statistics and the NHANES Program, we analyzed TCDD and TEQ concentrations in people who were 20–45 years of age in 2001–2002 (to compare against the similar age group in the 1970–1987 NHATS survey) or were born between 1936 and 1954 [47–66 years of age; i.e., of the same birth cohort as included in the study by Kang et al. (1990)]. We used Stata 9.0 (StataCorp, College Station, TX) to assess percentiles. For the NHANES data, we used the subsample-specific sampling weights provided in the NHANES data sets.

The second data set of more recent TEQ determinations comprises measurements taken in 2005 as part of the UMDES. The study was focused on assessing potential relationships between environmental dioxin exposures in the area of Midland and Saginaw Counties in Michigan, where elevated concentrations of PCDD/PCDF compounds have been identified in soils. The UMDES included a two-stage random sample of the population of Jackson and Calhoun Counties in Michigan (*n* = 251); these two counties constituted an external referent population, believed to be without unusual exposure to PCDD/PCDFs. This data set provides a key advantage over the NHANES data set because of the use of far larger serum volumes with the resulting increase in analytical sensitivity. The individual data were not available for analysis, but we used population-weighted summary data and statistics available on the study web site (UMDES 2008).

We calculated TEQ values for individual subjects in each study using the 1998 WHO TEFs for individual congeners (van den Berg et al. 1998) for PCDD/PCDFs and constructed box plots to describe interindividual variability in serum PCDD/PCDF TEQ within each data set using Stata 9.0. We used the 1998 TEF values because the response data sets evaluated here relied upon those TEF values (or an earlier version), and the original data were not available for recalculation.

### Dose–response characterization

The general approach used in this study was as follows: first, we assessed a quantitative relationship between the end point of interest and measured serum lipid-adjusted TEQ concentrations, and then we estimated a dose associated with a consistent benchmark response level across studies. For data sets addressing continuous variables, the benchmark response level was set at 10% extra risk of exceeding the normal range (as discussed, for example, by Gaylor and Aylward 2004). In the present study, we identified the limits of the normal range as the 2.5th and 97.5th percentiles in the general population, which corresponds to the typical delineation of clinical reference ranges (Siparsky and Accurso 2007). For data sets addressing quantal end points, the benchmark response level was likewise set to 10% extra risk of the event (BMD_10_). These BMD_10_ values can then be used as the basis of an assessment of MOE and changes in the MOE over time in the general U.S. population.

For this exploratory analysis, we selected three end points, each one based on data from a different study. The selection of these studies or end points does not represent a conclusion that a causal association between the exposure and the response has been established. End points were selected to be carried forward for quantitative analysis based on the biological relevance and plausibility of the end point examined, previous interest in the study and population, and as examples of various types of data that are found in the epidemiologic literature.

#### CYP1A2 activity

Lambert et al. (2006) followed a group of people highly exposed to PCBs and PCDFs due to accidental ingestion of contaminated rice oil in Taiwan (the Yucheng cohort). A total of 174 Yucheng and 134 control subjects were studied in an effort to determine the effectiveness of using induction of the CYP1 family of enzymes as a biomarker of exposure and effect in that cohort. Because CYP1A2 activity cannot be measured directly in humans, the caffeine breath test (CBT), a marker for CYP1A2 activity, was conducted. 3-*N*-Demethylation of caffeine is catalyzed by CYP1A2, so measurement of the proportion of exhaled ^13^C-labeled caffeine metabolites within a given time period (1 hr) after a known dose of ^13^C-labeled caffeine serves as a measure of that enzyme’s activity (Landi et al. 1999). Increasing enzyme activity (as reflected by increased caffeine metabolism rate) with increasing serum TEQ may represent aryl hydrocarbon receptor (AhR)-mediated induction of CYP1A2. These data constitute an evaluation of TEQ levels that can alter AhR-mediated gene expression in a human population as expressed by an early biochemical response known to be directly linked to AhR activation. Serum measurements included 17 PCDD/PCDFs and PCBs 77, 81, 126, 169, 105, 118, 156, 157, 167, and 189. Total dioxin TEQ was calculated based on the 1998 WHO TEFs (van den Berg et al. 1998). Lambert et al. (2006) presented a linear regression of percentage of ^13^C-labeled caffeine dose metabolized in an hour versus parts per trillion serum TEQ. A BMD_10_ for CYP1A2 induction was developed using this regression together with reported information on CBT variation among the controls.

#### Developmental defects of tooth enamel

Alaluusua et al. (2004) examined developmental defects of tooth enamel among subjects who were < 5 years of age (representing the age window during which development of permanent teeth occurs) at the time of exposure to TCDD after the explosion of a trichlorophenol production reactor in Seveso in 1976. The end point is of interest because a number of animal studies have demonstrated dental defects in rats exposed during the developmental period (reviewed by Alaluusua and Lukinmaa 2006) and because the Seveso population presents one of the few situations in which well-characterized exposure to TCDD has occurred in a non-occupational population. Dental examinations conducted 25 years after the accident were reported for 36 individuals who lived within the ABR zone (the area exposed during the accident) and 39 individuals who lived outside the ABR zone. Alaluusua et al. (2004) reported TCDD levels (but not levels of other dioxin congeners) for those subjects, based on serum that had been collected and frozen in 1976, and presented a categorical analysis by level of measured serum lipid TCDD in four exposure categories: non-ABR zone (background exposure), 31–226 ng/kg TCDD, 238–592 ng/kg TCDD, and 700–26,000 ng/kg TCDD. No individual measurements of other TEQ-contributing congeners were made in the Seveso serum samples, and no serum TCDD or TEQ measurements were available for the reference individuals. Therefore, we used the concentrations of 17 PCDD/PCDF compounds and nine TEQ-contributing PCB congeners measured in pooled serum samples for children 1–12 years of age collected during the 1970s period from outside the Seveso area as reported by Eskenazi et al. (2004). We assumed that Seveso children had similar serum levels of these non-TCDD congeners at the time of the accident and therefore estimated non-TCDD TEQ concentrations for Seveso residents as well as average total TEQ for the non-ABR reference individuals based on the data from Eskenazi et al. (2004). Because exposures are likely to be lognormally distributed, we took the antilog of the average of the log of the minimum and maximum of each categorical exposure range to represent a central estimate of exposure within each category. We conducted BMD modeling to estimate the serum lipid TEQ concentration corresponding to a 10% extra risk of dental defects using U.S. EPA Benchmark Dose Software [BMDS, version 1.4.1b (U.S. EPA, Washington, DC) with a variety of dichotomous models, with slopes restricted to be nonnegative to allow for the possibility of nonzero defect levels at zero exposure].

#### Thyroid hormone concentrations in infants

Koopman-Esseboom et al. (1994) examined thyroid hormone levels in 78 2-week-old infants from the general population in Rotterdam (the Netherlands) between 1990 and 1992 and related them to TEQ in their mothers’ milk (which was considered to represent lipid-adjusted TEQ of the mother and therefore a marker for *in utero* exposure levels). The mother–infant pairs were divided into two exposure groups: low (maternal milk TEQ less than or equal to the median, 72.43 pg TEQ/g lipid) and high (maternal milk TEQ > 72.43 pg TEQ/g lipid), and the mean and SE of infant free thyroxine (FT_4_) concentrations were reported for each group. Based on the reported mean, median, and SD of maternal TEQ concentration and assuming an overall lognormal distribution of TEQ concentrations in maternal milk, we estimated the mean milk TEQ concentrations in the lower and upper exposure groups and assumed a simple linear relationship between maternal milk TEQ and infant FT_4_ concentrations. We assume that the population variation in FT_4_ at any particular TEQ level is normally distributed with a degree of variability around its mean that is independent of TEQ and is equal to the average of the reported standard deviations of FT_4_ within each of the two exposure groups (low and high). The effect of TEQ on mean FT_4_ is calculated by the regression line between the high and low exposure groups. We assumed that maternal milk lipid-adjusted TEQ concentrations reflect maternal serum TEQ concentrations, an assumption that appears to be approximately correct, although some differential partitioning between breast and serum lipids occurs for higher chlorinated congeners (Wittsiepe et al. 2007). This differential partitioning likely has a limited effect on total TEQ in human serum and milk because of the relatively low contribution of higher chlorinated congeners.

### Characterization of changes in MOE

The MOE in a population is a unitless ratio between the POD and the estimate of dose or exposure in that population:


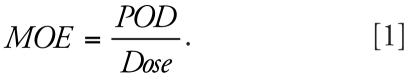


For this effort, both the POD and current exposure estimates are presented in terms of lipid-adjusted TEQ (WHO 1998) concentration. We also present an estimate of the current MOE, as well as a discussion of change in MOE from the 1970s era.

## Results

### Exposure

The adipose tissue samples analyzed by Kang et al. (1990) do not represent either a specific sampling year or specific age group but can generally be described as representing 1970s TEQ concentrations in males born between 1936 and 1954 (with a general age range at that time of 20–45 years). Descriptive statistics on the distribution of lipid-adjusted TEQ concentrations [using the WHO TEF values (WHO 1998)] are presented in Table 1 and Figure 2. No information on detection limits (LODs) was provided, and nondetects were included in the calculations as zero. Thus, the calculated TEQ concentrations may underestimate actual concentrations for this age group during this time period.

The results of the analysis of the NHANES data set for PCDD/PCDF TEQ are also presented in Table 1. Data were analyzed for two groups: *a*) adults 20–45 years of age at the time of the 2001–2002 sampling effort, and *b*) adults who were born between 1936 and 1954. These two groups correspond to the age range and birth cohort, respectively, included in the Kang et al. (1990) data set. Because the NHANES data set relied on very small serum sampling volumes, limits of detection (LODs) were quite high for many congeners. Ferriby et al. (2007) demonstrated that, particularly for individuals with relatively lower concentrations (for example, younger individuals), the choice of how to replace nondetected concentrations in TEQ calculations could affect estimated TEQs by 50–100%. We used two approaches, estimating percentiles under the assumption that nondetectable concentrations were zero (as used for the Kang et al. data set, for which no information on LODs was available) and assuming that nondetects were equivalent to the 
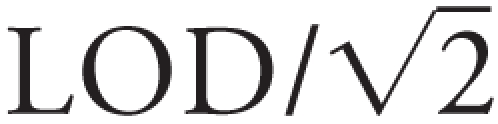
. For the younger age groups, choice of replacement for nondetectable concentrations results in nearly a 100% increase in the estimate of current median serum lipid concentrations. Upper-bound estimates are less sensitive to the assumption.

The data from the UMDES study reference population (Jackson and Calhoun counties, sampled in 2005) are available only in summary form for specific age groups (18–29, 30–44, 45–59, and ≥ 60 years). These age breakdowns allow a fairly direct comparison for the 20- to 45-year age group but are less useful for describing the concentrations in individuals born between 1936 and 1954 (51–69 years of age in 2005), particularly because of the substantial age-related changes in serum dioxin concentrations in persons >40 years of age (Ferriby et al. 2007). Summary statistics for the relevant overlapping age groups from the UMDES study are also presented in Table 1. Because substantial serum volumes were available for all analyses, TEQ estimates are not affected substantially by the presence of nondetectable concentrations.

Figure 2 presents box plots comparing the 1970s data with the current data sets for persons 20–45 years of age during both time periods (Figure 2A) and for persons born during the 1936–1954 period over time (Figure 2B). Within individuals born between 1936 and 1954, both median and 95th percentile PCDD/PCDF TEQ concentrations between the 1970s-era sampling and the NHANES 2001–2002 sampling years decreased by approximately 75%. Serum lipid TEQ concentrations in persons 20–45 years of age in the 2001–2002 NHANES data set decreased by approximately 85% compared with the 1970s-era sampling, a difference reflected in both the medians and 95th percentiles. These comparisons do not include PCB contributors to TEQ, but a comparison of wet-weight serum PCB concentrations observed in studies conducted in the 1970s (Kreiss 1985) with results from the NHANES 2001–2002 data sets (Nichols et al. 2007) suggests a similar or larger magnitude of decline in serum PCB concentrations over the same period, although no statistically representative data sets are available to specifically assess changes in TEQ-contributing PCB congener concentrations over that period.

The decrease in serum lipid TEQ observed among persons born between 1936 and 1954 indicates that ambient exposures have declined substantially since the 1970s. Because the elimination of PCDD/PCDF compounds occurs with half-lives on the order of 5–10 years, declines in serum lipid TEQ will continue for many years after decreases in exposure levels (Aylward and Hays 2002; Lorber 2002). As a result, serum TEQ concentrations in the population in the future will likely decline further, and evaluations of MOE for the purposes of assessing potential risks from current intake rates should be based on measured concentrations in younger individuals with the least impact from historically higher exposure levels. As discussed above, the UMDES study appears to provide the most reliable data set for characterizing current serum concentrations in young adults because of the very low LODs attained in that study. The median and 95th percentile total TEQ [according to the WHO 1998 TEF scheme (WHO 1998), including PCB contributors to TEQ] reported for the UMDES study referent population 18–29 years of age based on 2005 sampling are 9.2 and 13.3 ppt TEQ, respectively. These concentrations are used below to characterize current MOE in the young adult population of reproductive age in the United States, a population of high interest for risk assessment of dioxins.

### Response benchmark characterization

#### CYP1A2 induction

Lambert et al. (2006) presented a linear regression of percentage of ^13^C-labeled caffeine dose metabolized in an hour versus parts per trillion serum TEQ [WHO 1998 TEQ (WHO 1998)] with a reported slope of 0.0029 and an intercept of 1.17% of dose metabolized. An estimate of the pooled (male and female) weighted average and SD of CBT in the 134 controls can be derived from data (1.18% ± 1.20) of the CBT in male and female controls of Lambert et al. (2006). If the distribution of CBT is normal, then the 97.5th percentile of the amount metabolized in 1 hr is approximately 3.5%; this appears to be generally consistent with the data presented in graphical form by Lambert et al. (2006).

**Figure 1:**
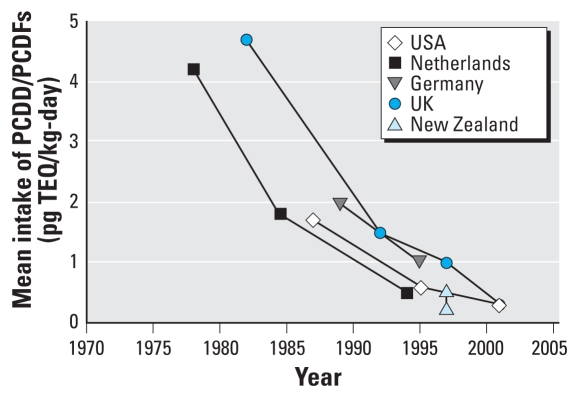
Estimated dietary intakes of PCDD/PCDFs in the United States (U.S. EPA 2000; U.S. FDA 2008), the Netherlands (Liem et al. 2000), the United Kingdom (UKFSA 2003), Germany (Furst and Wilmers 1997), and New Zealand (Smith and Lopipero 2001). New Zealand data represent a range of estimates based on central and upper-end intake and concentration estimates. Adapted from Hays and Aylward (2003).

**Figure 2:**
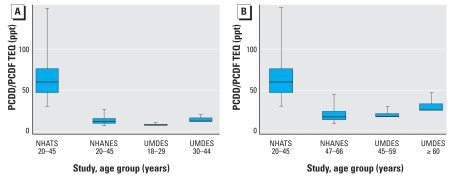
Box plots illustrating the distribution of measured PCDD/PCDF TEQ (WHO 1998) concentrations in samples from individuals age 20 to 45 years of age (*A*) or born between 1936 and 1954 (*B*) in the general population in the United States. Boxes represent the interquartile range (25th–75th percentiles; median indicated by horizontal line), and whiskers extend to the 5th and 95th percentiles. NHATS, lipid-adjusted adipose tissue concentrations from NHATS samples collected between 1971 and 1982 (Kang et al. 1990); NHANES, lipid-adjusted serum concentrations from the NHANES 2001–2002 survey assuming nondetects are present at 
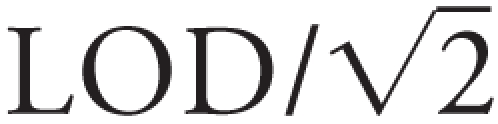
; UMDES, lipid-adjusted serum concentrations from samples collected in 2005 from residents of referent counties in Michigan (UMDES 2008); 5th and 25th percentiles were not reported from this study and so are not included in the box plots here.

Assuming that this degree of inter-individual variability applies at any specific dioxin serum level [as appears reasonable from examination of Lambert et al.’s Figure 1 (Lambert et al. 2006)], then a BMD_10_ can be calculated as the serum TEQ necessary to shift the distribution such that an additional 10% of the population has a CBT exceeding 3.5%. Using the regression equation calculated by Lambert et al. (2006) and the assumption of normal variability in the CBT outcome around its mean value yields an estimate of serum TEQ BMD_10_ of approximately 340 ng/kg lipid. Assumption of lognormal distribution of values around the mean results in a BMD_10_ estimate of approximately 400 ppt TEQ.

#### Dental defects

Table 2 presents the prevalence of dental aberrations, the measured TCDD and estimated serum lipid TEQ ranges, and estimated midpoint TEQ concentrations, by category, from Alaluusua et al. (2004). The results of BMD modeling for the data set are presented in Table 3. The log-logistic model produced the best fit to the data [i.e., the lowest Akaike information criterion (AIC) value], and three of the models resulted in slightly higher BMD_10_ estimates.

#### Thyroid hormone concentrations

**Figure 3:**
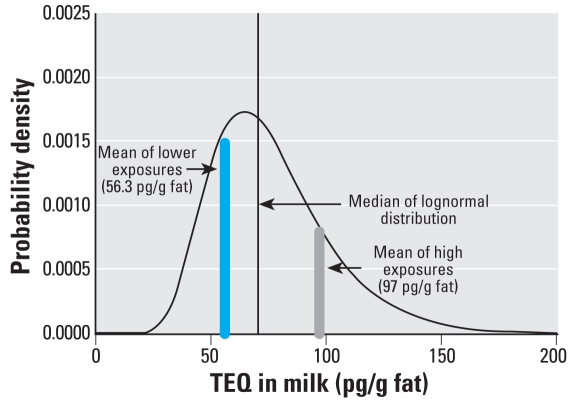
Estimated probability distribution of lipid-adjusted human milk concentrations from Koopman-Esseboom et al. (1994) based on summary statistics discussed in the text.

For the dose–response analysis for FT_4_ from Koopman-Esseboom et al. (1994), we estimated mean exposures for both exposure groups. Koopman-Esseboom et al. (1994) reported maternal milk TEQ concentrations using the 1993 WHO TEF values. However, the changes in the WHO TEF values between 1993 and 1998 result in offsetting changes in estimates of TEQ (approximately a 15% increase in estimates of PCDD/PCDF TEQ, and a similar decrease in PCB TEQ estimates at the mean because of the changes in TEF values between the two versions of the TEF system). Thus, the TEQ concentrations reported by Koopman-Esseboom et al. (1994) are used directly in this analysis, on the assumption that the WHO 1998 TEQ (WHO 1998), if it could be calculated from the reported data, would be expected to be similar. Because the mother–infant pairs were drawn from the local population without prior knowledge of exposure, we assumed a lognormal distribution. Using the arithmetic mean (74.86 pg TEQ/kg lipid) and SD (26.19) for milk TEQ reported for the overall cohort, we calculated a geometric mean (70.66 pg TEQ/kg lipid) and geometric SD (1.405). This estimated geometric mean corresponds well with the reported median of 72.43 pg TEQ/kg lipid, and the lognormal assumption predicts the observed range of the milk TEQ values well. Assuming this lognormal distribution for the overall cohort, mean TEQs for the low- and high-exposure groups were estimated to be 56.3 pg TEQ/g lipid and 97 pg TEQ/g lipid, respectively, as illustrated in Figure 3.

Assuming that the relationship between FT_4_ and milk TEQ is linear, the two calculated mean exposure levels and corresponding means of the measured FT_4_ concentrations for the low- and high-exposure groups define a regression line; increasing levels of TEQ were associated with decreasing levels of FT_4_. The slope of the regression line is –0.0392 pmol/L per 1 pg TEQ/g lipid in milk, and the intercept is 26.8 pmol/L.

For a given TEQ there will be natural variation in FT_4_ levels among infants. SDs for measured FT_4_ concentrations were given by Koopman-Esseboom et al. (1994) as 3.5 pmol/L for the low-exposure group and 3.3 pmol/L for the high-exposure group. The BMD_10_ is defined in this case as the TEQ exposure that would change the mean FT_4_ such that an additional 10% of the variable population drops below the FT_4_ level that constitutes the 2.5th percentile in an unexposed population (with zero exposure). To do this, we assume that the population variation in FT_4_ at each exposure level is normally distributed with a mean calculated by the regression line and a standard deviation calculated as the average of the standard deviations for the two measured data points, namely (3.5 + 3.3)/2 = 3.4 pmol/L. We assume further that the variability around the regression is independent of TEQ.

At an exposure level of zero TEQ, the mean FT_4_ is estimated to be 26.8 pmol/L (the regression line intercept), and using the observed SD of 3.4 pmol/L (mean of the two provided SDs), the 2.5th percentile FT_4_ level is 20.1 pmol/L. This value is consistent with published neonatal thyroid hormone reference ranges (Hubner et al. 2002). The BMD_10_ is then the TEQ exposure at which an extra 10% of the population has FT_4_ concentrations ≤20.1 pmol/L, which occurs when the mean FT_4_ concentration is 24.0 pmol/L and corresponds to a BMD_10_ of approximately 70 pg TEQ/g lipid in milk.

### MOE characterization

The BMD_10_ values calculated for each end point are presented in Table 4. These values are best estimates from the specific studies and data sets using the assumptions outlined in this manuscript and are provided as examples of potential quantitative approaches to a variety of epidemiologic data sets that are available. Table 4 also provides an overview of the estimated margins of exposure for each of the end points based on estimates of current serum lipid TEQ concentration (including PCDD/PCDF and PCB compounds) in young adults at the median and at the 95th percentile. MOEs for the same end points in the 1970s would have been approximately six to seven times lower, based on the comparative exposure data summarized above.

## Discussion

### Consideration of target MOE

The MOE estimates presented in Table 4 can be compared with a target minimal MOE related to the usual application of uncertainty factors in the derivation of reference doses, minimal risk levels, and other benchmarks for general population environmental exposures. Table 5 outlines the typical uncertainty factors applied and provides a discussion of the applicability of such factors to a risk assessment based on human data using an internal measure of biologically relevant exposures. Selection of a target MOE requires a judgment regarding the biological relevance or adversity of the modeled benchmark response, so that larger or smaller components of the lowest observed adverse effect level (LOAEL) to no observed adverse effect level (NOAEL) uncertainty factor might be selected for a given end point or selected benchmark response level (e.g., a 10% increase in the proportion of the population exhibiting increased enzyme activity might be considered a less adverse benchmark than a 10% increase in the proportion exhibiting decreased FT_4_ concentrations). Because both the dose–response assessment and the exposure assessments for this evaluation are based upon biologically relevant internal dose metrics (circulating serum TEQ concentration), the uncertainty factor component typically applied for intraspecies toxicokinetic differences is replaced by the actual sampling data in the population, which explicitly reflects the variations in toxicokinetics among individuals. That is, individuals who are pharmacokinetically sensitive (presumably those who eliminate the compounds more slowly) directly reflect this sensitivity in the measured serum lipid concentrations, which will be higher than those in the less-sensitive members of the population.

The current MOEs estimated for CYP1A2 and for dental defects are generally above the range of target minimal MOEs, suggesting that these end points may not be of concern at current background serum concentrations. However, the current MOEs for thyroid hormone alterations in infants based on the Koopman-Esseboom et al. (1994) data and current U.S. exposure data are at the lower end of the range of target MOE values, although there is a clear separation between current U.S. concentrations in young adults of reproductive age and the BMD_10_ for this end point. That is, the current upper-bound TEQ concentration in young adults of reproductive age in the United States (~ 14 ppt TEQ) is approximately 5-fold lower than the BMD_10_. Recent studies of this end point provide conflicting results: Maervoet et al. (2007), in a study of 140 Flemish infants, report an association between maternal serum TEQ and umbilical cord FT_4_ of similar magnitude, whereas Wilhelm et al. (2008) report no relation between maternal milk or blood TEQ and cord blood thyroid hormone concentrations in a smaller study of German infants. Other researchers have also found conflicting results [reviewed by Giacomini et al. (2006) and Maervoet et al. (2007)]. The high degree of correlation between serum TEQ and non–TEQ-contributing organochlorine compounds complicates the identification of causal agents and relationships. Other factors not typically controlled for in such studies, such as gestational age and serum lipid concentration, may also confound the relationships observed (reviewed by Maervoet et al. 2007).

The BMD_10_ estimates presented here in terms of serum lipid TEQ can be compared with the adipose tissue concentrations reported in key animal studies previously used to derive the JECFA (2001), UKCOT (2001), and ECSCF (2001) tolerable daily intakes. Maternal wet-weight adipose tissue concentrations in the key studies at the LOAELs or NOAELs can be estimated as ranging from approximately 65 to 200 ppt TCDD (Faqi et al. 1998; Hurst et al. 2000). If these wet-weight concentrations are adjusted assuming 80% lipid content of adipose tissue, these correspond to approximately 80–250 ppt TEQ, comparable to the range of BMDs derived here (Table 4).

The assessment of current exposures based on measured serum lipid concentration has several strengths over conventional exposure assessments based on intake. Such measurements aggregate exposure from all exposure routes and media, which individually may be difficult to quantify because of low concentrations. Reliance on measured serum concentrations also reduces uncertainties in risk assessment regarding potential interindividual pharmacokinetic differences, because such differences are directly reflected in the measured concentrations. Finally, circulating serum lipid concentration is likely to be a good surrogate for critical target tissue exposure concentrations in a range of organs and tissues because of the lipophilic nature of dioxin-like compounds.

When interpreting these results, some limitations inherent in the use of measured TEQ concentrations for exposure quantification should be borne in mind. The TEF system was developed based on relative potencies on the basis of intake, not tissue concentration. Thus, differential toxicokinetics among congeners can lead to distortions in the estimate of total toxic equivalency when TEQ is assessed on a tissue concentration basis rather than an intake basis. Such distortions might be of limited concern when the dose–response assessment is conducted on populations with similar distributions of congeners as the current background population. However, for two of the data sets used here, the data from Lambert et al. (2006) on CYP1A2 activity and the Seveso data on dental defects (Alaluusua et al. 2004), the TEQ tissue concentrations resulted from unusual exposures to congeners or mixtures that are not reflective of background exposure patterns, and thus extrapolation of responses observed in those populations to background exposures on a serum TEQ concentration basis may pose additional uncertainties.

Interpretation of results from epidemiologic studies must proceed cautiously because of the complexities inherent in such studies: cross-sectional epidemiologic studies are not analogous to toxicologic experiments, and data from single studies cannot be interpreted in isolation. Numerous factors outlined by Hill (1965), including consistency of evidence across studies, biological plausibility, and accounting for potential confounding, must be assessed. The three examples presented here represent data sets on end points with varying degrees of consistency in the epidemiologic literature. Induction of CYP1A2 activity in humans has been observed previously in individuals with highly elevated exposures [reviewed by Connor and Aylward (2006)], and the magnitude of dose response reported by Lambert et al. (2006) is consistent with previous reports. The pattern of evidence related to potential impacts of dioxin-like compounds on infant thyroid hormone levels is less consistent, as discussed above. The dental anomalies reported by Alaluusua et al. (2004) in Seveso children are biologically plausible based on results from studies in rodents, and data from the Seveso study and other studies support a conclusion that exposures above current background exposures would be required to induce such effects (Alaluusua and Lukinmaa 2006).

The data sets used to characterize dose response in this exercise are typical of many epidemiologic studies in that data are not generally presented in a framework that allows direct application of quantitative risk assessment methodologies. Several common epidemiologic methods used in such studies pose challenges in the risk assessment context:

- When exposures and responses are presented in categorical analyses [for example, in the analyses by Koopman-Esseboom et al. (1994) and Alaluusua et al. (2004)], evaluation of dose response and identification of BMDs require numerous assumptions regarding representative exposures in each category. For example, in the assessment of the dental defect data for Seveso, we estimated the median TCDD concentration in each exposure category based on an assumption of an overall lognormal distribution of exposures. However, other assumptions could have been used, and the estimated BMD values would change accordingly.
- In continuous analyses, full regression results are often not reported, making it difficult to estimate BMDs at appropriate values of important covariates. In addition, such regressions typically impose a shape on the fitted dose–response curve, including an assumption of linearity that extends to the lowest exposure levels. Because the slope of a linear regression is often dominated by responses at the higher end of exposures, the possibility of a zero slope at lower exposures (in effect, the existence of a threshold for the response) is masked in the regression.
- Characterization of the interindividual variability in the response parameter of interest, including description of the distribution type (normal, lognormal) and the magnitude of typical variability, is often lacking. In particular, information sufficient to evaluate interindividual variability in a parameter in the absence of the influence of the exposure of interest is generally not provided.
- In many epidemiologic studies, results are reported as odds ratios. Conversion of relationships reported in this way into continuous functions that can be used to estimate BMDs can be difficult or impossible, depending upon what other information is provided.
- Similarly, epidemiologic studies often report results using analyses based on logarithmically transformed exposure measures. Such results are difficult to extrapolate beyond the range of the experimental data with any confidence. For example, such dose–response functions imply that a change in exposure from 0.1 to 0.3 ppt TEQ might be associated with the same magnitude of response as an increase in exposure from 100 to 300 ppt, which may not be biologically plausible. When attempting to estimate background responses, such functions may not be appropriate for extrapolation from higher exposures.

A comprehensive risk assessment effort based on the available human data sets would require a systematic assessment of the consistency and weight of the evidence and human relevance of each end point before quantitative analysis of the data sets was conducted. Quantitative analysis could then proceed on end points with strong or moderate levels of evidence to provide a full quantitative context for interpretation of exposure data.

The analysis presented here demonstrates that young adults in the U.S. population have current serum TEQ concentrations ≥ 6-fold lower than adults of the same age in the 1970s. Thus, the margins of exposure for all potential effects of dioxin-like compounds have increased by a factor of six over the past three decades. Because a substantial body of epidemiologic studies of potential health impacts of dioxins relying upon measured serum TEQ as the exposure metric now exists, such exposure data can be used directly in the risk assessment arena to assess margins of exposure for responses of interest. Some uncertainties arise in using such data in a risk assessment context because of limitations in reporting exposure or response parameters in such studies. However, application of risk assessment methodologies to this body of epidemiologic data avoids uncertainties inherent in interspecies extrapolation and in accounting for pharmacokinetic variability that may result in differential impacts of a given estimated intake dose.

## Figures and Tables

**Table 1:** Median (95th percentile) of measured lipid-adjusted PCDD/PCDF TEQ concentrations from sampling conducted in the United States in the 1970s and 2001–2005.

	WHO 1998 TEQ, PCDD/PCDF		
	Sampling year(s)	ND = 0	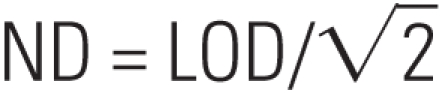
Birth cohort, 1936–1954			
NHATS	1971–1982	60.2 (151.1)	
NHANES	2001–2002	12.4 (43.5)	17.2 (44.5)
UMDES referents, birth year < 1940	2005		25.7 (46.7)
UMDES referents, birth years 1940–1960	2005		17.6 (30.1)
Adults 20–45 years of age			
NHATS	1971–1982	60.2 (151.1)	
NHANES, ages 20–45 years	2001–2002	5.9 (22.4)	11.7 (26.7)
UMDES referents, ages 18–29 years	2005		7.0 (9.7)
UMDES referents, ages 30–44 years	2005		12.2 (20.2)

ND, nondetects. NHATS data from Kang et al. (1990).

**Table 2:** Developmental defects of enamel in individuals who were children (< 5 years of age) at the time of the Seveso accident, referents, and Seveso residents by tertile.

Exposure group (*n*)	Range of serum TCDD (ng/kg lipid)	Estimated median TCDD concentration (ng/kg lipid)^a^	Estimated TEQ (ng/kg lipid)^b^	Prevalence (%)
Non-ABR subjects (39)	NM	40.5	116.6	10 (26)
Seveso				
1st tertile (10)	31–226	83.7	159.8	1 (10)
2nd tertile (11)	238–592	375.4	451.5	5 (45)
3rd tertile (15)	700–26,000	4266.1	4342.2	9 (60)

NM, not measured. Data from Alaluusua et al. (2004)

a Assumes lognormal distribution of serum TCDD measurements (see “Methods”).

b Estimated based on data from Eskenazi et al. (2004). Average non-TCDD TEQ (16 PCDD/PCDF and 9 PCB compounds) measured in two pooled serum samples from children 1–12 years of age from outside Seveso collected during the same time period was 76.1 ng TEQ/kg lipid; this value was added to estimated median TCDD values for each Seveso exposure group. Total TEQ (including background TCDD) averaged 116.6 ng/kg TEQ in the two pooled samples; this value was used as the TEQ concentration for the non-ABR referents.

**Table 3:** BMD_10_ modeling results for dental defects.

Model	BMD_10_	BMDL_10_	AIC
Gamma (power ≥1)	666.2	330.8	93.1582
Log-logistic (slope ≥1)	445.3	140.1	92.9532
Multistage (1-degree, beta ≥0)	666.2	330.8	93.1582
Probit (slope ≥1)	1296.9	636.3	93.6437
Weibull (power ≥1)	666.2	330.8	93.1582

BMDL_10_, statistical lower bound on the BMD_10_. Data from Alaluusua et al. (2004).

**Table 4:** Summary of modeled BMD_10_ values and estimated MOEs at the median and upper bound of current lipid-adjusted TEQ concentrations in young adults of reproductive age in the United States for three end points based on example data sets.

		MOE^a^	
End point	BMD_10_ (ng TEQ/kg lipid)	At median current U.S. TEQ (9.2 ppt)	At 95th percentile current U.S. TEQ (13.3 ppt)
CYP1A2 activity	340	35	25
Dental defects	450–1,300^b^	50–140	30–95
Neonatal FT_4_ changes	70	8	5

a Compared with current median and 95th percentile exposures as estimated by UMDES reference population 18–29 years of age, 29 PCDD/PCDF and PCB congeners, and the WHO 1998 TEQ.

b Range of BMD_10_ estimates from different BMD models.

**Table 5:** Evaluation of typical uncertainty factor components in the context of identification of target minimal MOE for risk assessments based on human studies employing serum lipid TEQ concentrations as the exposure metric.

Uncertainty factor component	Typical value	Applicable?	Target value
LOAEL to NOAEL	≤ 10	Data-dependent. Depending on the benchmark chosen, the POD may be regarded as more similar to a NOAEL or a minimal or frank LOAEL.	Varies by end point 1–10
Interspecies	10	No. Risk assessment based on human data.	1
Intraspecies PK	3	No. Interindividual variations in pharmacokinetics are directly reflected in measured serum lipid TEQ concentrations. Pharmacokinetically “sensitive” individuals will manifest higher measured concentrations, so the sensitivity is reflected directly in the exposure assessment.	1
Intraspecies PD	3	Yes, although if the study used as the basis of the BMD determination includes the sensitive subpopulation, this value may be reduced.	1–3
Target MOE			1–30

Abbreviations: LOAEL, lowest observed adverse effect level; NOAEL, no observed adverse effect level; PD, pharmacodynamic; PK, pharmacokinetic.
